# Integrating Phenotypic Information of Obstructive Sleep Apnea and Deep Representation of Sleep-Event Sequences for Cardiovascular Risk Prediction

**DOI:** 10.21203/rs.3.rs-4084889/v1

**Published:** 2024-03-15

**Authors:** Yali Zheng, Zhengbi Song, Bo Cheng, Xiao Peng, Yu Huang, Min Min

**Affiliations:** Shenzhen Technology University; Shenzhen Technology University; Shenzhen Technology University; Shenzhen Technology University; Shenzhen Technology University; Sun Yat-sen University

**Keywords:** Cardiovascular risk prediction, obstructive sleep apnea phenotyping, deep representation, sleep event sequences, phenotype-aware models, model interpretability

## Abstract

**Background:**

Advances in mobile, wearable and machine learning (ML) technologies for gathering and analyzing long-term health data have opened up new possibilities for predicting and preventing cardiovascular diseases (CVDs). Meanwhile, the association between obstructive sleep apnea (OSA) and CV risk has been well-recognized. This study seeks to explore effective strategies of incorporating OSA phenotypic information and overnight physiological information for precise CV risk prediction in the general population.

**Methods:**

1,874 participants without a history of CVDs from the MESA dataset were included for the 5-year CV risk prediction. Four OSA phenotypes were first identified by the K-mean clustering based on static polysomnographic (PSG) features. Then several phenotype-agnostic and phenotype-specific ML models, along with deep learning (DL) models that integrate deep representations of overnight sleep-event feature sequences, were built for CV risk prediction. Finally, feature importance analysis was conducted by calculating SHapley Additive exPlanations (SHAP) values for all features across the four phenotypes to provide model interpretability.

**Results:**

All ML models showed improved performance after incorporating the OSA phenotypic information. The DL model trained with the proposed phenotype-contrastive training strategy performed the best, achieving an area under the Receiver Operating Characteristic (ROC) curve of 0.877. Moreover, PSG and FOOD FREQUENCY features were recognized as significant CV risk factors across all phenotypes, with each phenotype emphasizing unique features.

**Conclusion:**

Models that are aware of OSA phenotypes are preferred, and lifestyle factors should be a greater focus for precise CV prevention and risk management in the general population.

## 1. INTRODUCTION

Cardiovascular diseases (CVD) are the leading cause of death globally. According to the World Health Organization, the annual deaths from CVDs exceed 17 million, accounting for approximately 32% of all mortalities [1]. Risk prediction is crucial for the prevention and treatment of CVDs. Traditionally, medical experts make qualitative predictions based on experience, supplemented by statistical models such as Framingham Risk Score [2], SCORE [3], and Cox Proportional Hazard [4]. Recently, Machine Learning (ML) and Deep Learning (DL) techniques have emerged as powerful tools for enhancing CVD risk prediction [5–11].

Despite these advances, many studies have not fully considered the complex interplay of CVD risk factors with concurrent clinical conditions. There is a recognized link between Obstructive Sleep Apnea (OSA) and increased CVD risk, with OSA prevalence in CVD patients ranging from 40% to 80% [12]. Recent research has focused on OSA phenotyping using polysomnography (PSG) data, identifying distinct CVD risk levels among different OSA phenotypes [13–17]. The pathophysiology of OSA-related cardiovascular complications has been recognized as multifaceted, encompassing four main domains: sleep architecture disturbances, autonomic dysregulation, breathing disturbances, and hypoxemia [18–21]. However, existing research have not integrated the OSA phenotypic information with traditional risk factors in predictive modeling, leaving the actual predictive effectiveness uncertain.

On the other hand, the increasing ease of gathering long-term physiological data through wearable and mobile devices, combined with the advanced capabilities of deep learning (DL) in complex signal representation, has spurred studies integrating these signals into CV risk prediction. For example, Dami et al. developed a DL model to predict arterial events over a course of a few weeks/months, which outperformed traditional machine learning (ML) and other DL approaches [22]. They applied a Deep Belief Network (DBN) to select effective features from *5-minute* ECG recordings, and then made predictions with a long short-term memory (LSTM) network in conjunction with features from the electronic health records (EHRs). Chen et al. employed LSTM to learn deep representations from multi-channel physiological signals. By combining these with static features, they then used a Gradient Boosting Machine (GBM) to predict six types of adverse events in the next 5 minutes of surgery with promising results [23]. Yet, existing studies [24–27] have mainly focused on the prediction of specific CV events and over relatively short period of time. There is limited research about utilizing extended periods of physiological signals for long-term CV risk assessment. Considering the advancements in wearable and mobile technologies, the integration of EHR information and daily health data for long-term CVD risk prediction holds significant potential benefits for cardiovascular healthcare.

Therefore, this study aims to explore effective methods to incorporate OSA phenotypic information and leverage extended periods of physiological data for CV risk prediction over long-term periods. And, a feature importance analysis is conducted to identify the most significant risk features in the general population and across different OSA phenotypes to provide insights for precise CV risk management. The main contribution of this study is summarized as follows:

1. Proposed a novel and effective method for precise long-term CV risk prediction, by integrating deep features learned from overnight physiological sequences and employing an OSA-phenotype-contrastive training strategy;
2. Identified lifestyle-related features as significant CV risk factors among a comprehensive feature set, and revealed distinct risk features across different OSA phenotypes, offering new insights for precise management of CV risks.

## 2. METHODS

### 2.1 Datasets and Subjects

This study utilized data from the Multi-Ethnic Study of Atherosclerosis (MESA), initiated and collected by the National Heart, Lung, and Blood Institute (NHLBI) [28,29]. MESA study recruited a total of 6,814 participants without CVDs aged between 45 to 84 years from four ethnic groups (African Americans, Chinese Americans, Hispanics, and Caucasians). Five examinations were conducted between 2000 and 2011, and clinical outcomes were assessed every 9 to 12 months during this period, including: myocardial infarction, angina, heart failure, coronary heart disease, and death.

During Exam_5, 2,237 participants of the MESA population participated in an auxiliary sleep study, and 2,489 static features were recorded, including demographics, anthropometrics, medication usage, medical history, imaging risk factors, etc. Among them, 1,874 participants had provided overnight PSG data, which includes 27 continuous physiological signals recorded during sleep, including electrocardiography, electroencephalography, pulse, nasal airflow, blood oxygen, periodic limb movements (PLMS), and more. The average recording duration was 12 hours per participant. Additionally, 615 sleep-related static features were extracted from the sleep questionnaires, actigraphy-derived parameters and average counts of overnight sleep events derived from PSG recordings (so there are in total of 3,104 static features). Manual annotations were performed on the PSG data for identifying sleep stages and events (such as arousal, hypopnea, apnea, hypoxemia, periodic limb movements). This study mainly focused on the analysis of the 1,874 participants, who have complete sets of static features and PSG recordings, and 175 of them (9.3%) experienced CVD events. The whole dataset was divided into training and validation sets, comprising 1,687 and 187 subjects, respectively. The validation set proportion is 9.3%.

### 2.2 Study Pipeline

**Figure 1:**
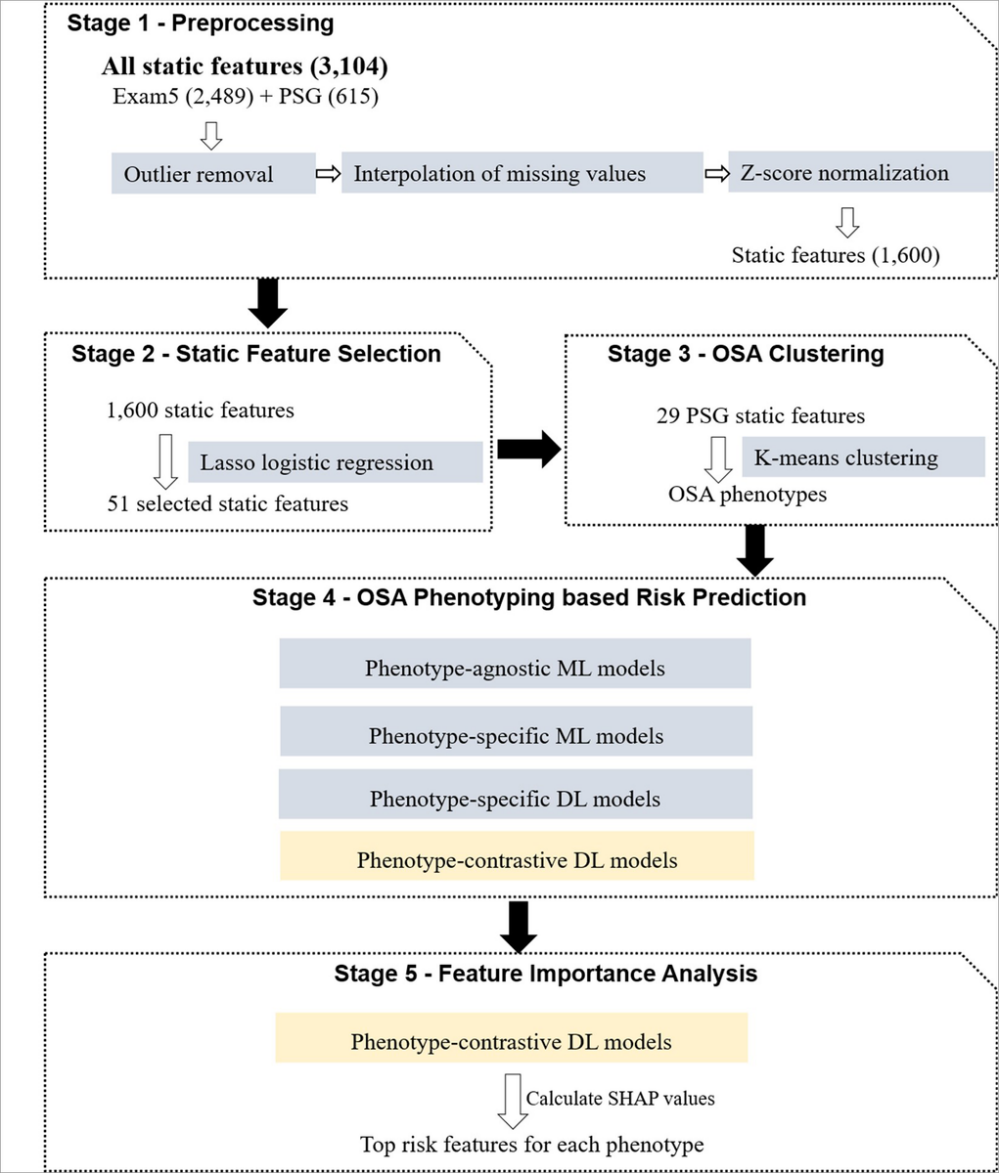
The overview of the study pipeline

Initially, feature selection was performed on all static features by lasso-logistic regression. Clustering was then conducted based on the overnight average values of PSG features (named PSG static features) to identify OSA phenotypes. Subsequently, several ML and DL models with different feature selection and training strategies were built to explore the most effective method of incorporating OSA phenotypic information for CVD risk prediction. Finally, the feature importance analysis was conducted for each phenotype based on the best performing model. The study pipeline is shown in Fig. 1.

#### 2.2.1 Data Preprocessing

The following preprocessing steps were applied to the 3,104 static features (2,489 exam_5 static features and 615 PSG static features) of all participants. First, data outliers were removed, including blank values, duplicates, and irrelevant numerical values. Missing values were then imputed with mean interpolation. Finally, z-score normalization was applied to standardize each feature. The preprocessing process resulted in each participant having 1,600 normalized static features.

**Figure 2:**
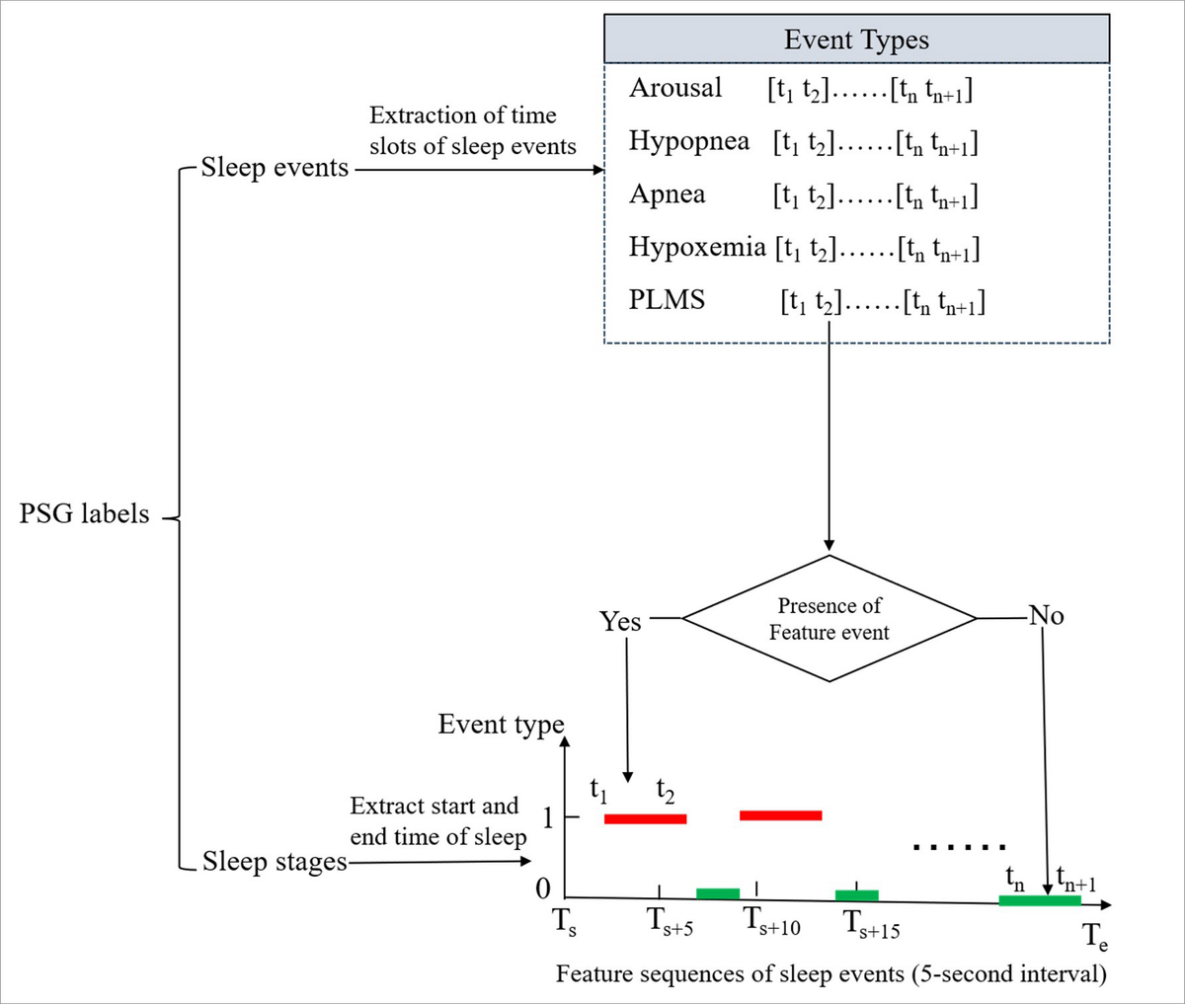
Generation of the overnight feature sequences of the five sleep events, including: arousal, hypopnea, apnea, hypoxemia and PLMS. Ts: start time of sleep, Te: end time of sleep

Considering our goal is to predict 5-year CVD risk, while multiple high-fidelity physiological signals exhibit highly complex temporal patterns throughout the entire sleep period, directly using them as model inputs may not capture efficient and effective information related to long-term CVD risks. Therefore, this study chose to learn deep representations from the feature sequences of five sleep events known contributed to CVD risks, including arousal, hypopnea, apnea, hypoxemia and PLMS. The sequences of the five sleep events were generated according to the PSG labels through the following steps. Firstly, sleep sequences were generated with a 5-second sampling interval during the start and end sleeping time of each participant. Then, the time slots of the occurrences of each sleep event were extracted from the PSG labels to determine whether the event occurred within any of the 5-second sampling intervals. These events were marked as '1' if they occurred within an interval, and '0' otherwise. This created five overnight sleep-event sequences for further analysis. (Fig. 2) illustrates the generation process of the overnight feature sequences of five sleep events.

#### 2.2.2 Static feature selection

Lasso logistic regression was used to select a subset of features from the 1,600 static features using the training set. Ten-fold cross validation was conducted to determine the regularization hyperparameter, alpha. It was adjusted across a range of values: 0.0001, 0.0005, 0.001, 0.005, 0.01, 0.015, 0.02 and 0.025, to identify its optimal value that yields the best predictive performance on the validation set.

#### 2.2.3 OSA clustering

29 PSG static features (the features shown in Supplementary Table 2) were employed for OSA clustering utilizing the K-means clustering. A range of 2 to 6 clusters was explored to determine the optimal cluster number by the silhouette and elbow methods.

Mann-Whitney U tests were then conducted on the 29 PSG static features between different OSA phenotypes to identify the most relevant PSG features for each phenotype. A significance level below 0.05 was considered as significantly different, while a *P* value below 0.001 was considered highly significant.

Cox proportional hazards regression analysis was also performed to estimate the hazard ratio (HR) and 95% confidence intervals (CI) of occurring CVD events within five years for different OSA phenotypes.

#### 2.2.4 OSA phenotyping-based CVD risk prediction modeling

To assess the value of incorporating OSA phenotypic information in CVD risk prediction, several classic ML models were employed in this study, including logistic regression (LR), support vector machine (SVM), decision tree (DT), random forest (RF), gradient boosting machine (GBM) and multilayer perceptron (MLP). The phenotype-agnostic method did not consider any phenotypic information. For phenotype-specific models, two different strategies to integrate the phenotypic information and three different feature sets were evaluated. The details of the phenotype-specific ML models are described as below:

1. *Pheno_fuse_ML*: The OSA phenotyping labels (Phenotypes 1, 2, 3, 4) were incorporated as a new feature along with selected static features, serving as input for the ML models to predict CVD risk across the entire population.
2. *Pheno_specific_ML*: The strategy develops CVD risk prediction models specific to each phenotypic population, using the three different sets of features:
  1. *Pheno_Spec_ML1*: Uses only selected static features.
  2. *Pheno_Spec_ML2*: Combines the 29 PSG static features with the selected static features.
  3. *Pheno_Spec_ML3* Fuses phenotype-specific static PSG features representing each phenotype with the selected static features.

**Figure 3:**
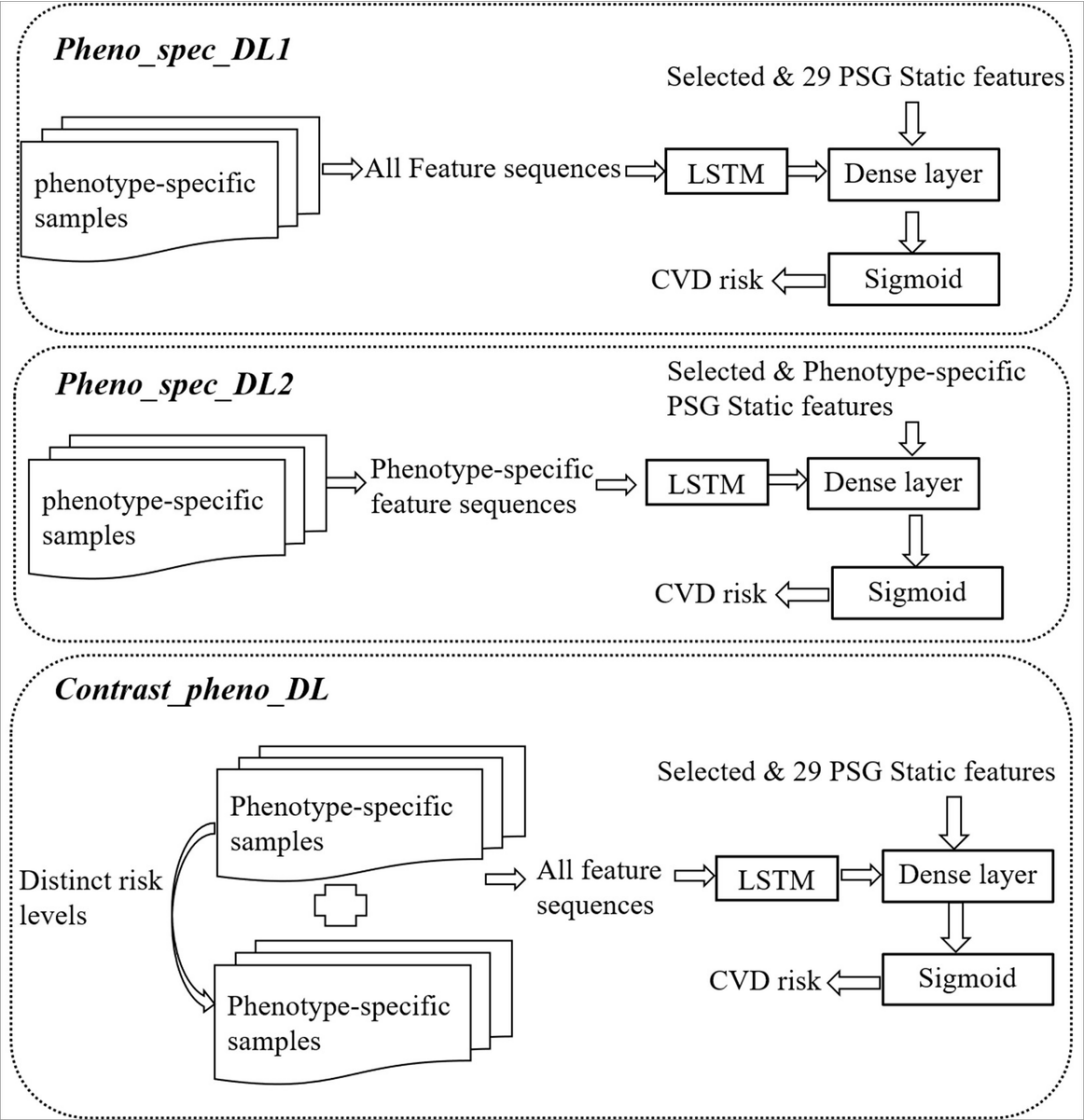
The proposed OSA phenotyping based deep learning model with different training strategies and feature sets for CVD risk prediction.

To further explore the value of overnight sleep-event feature sequences in CVD risk prediction, a two-layer LSTM network was employed to learn deep representations from the overnight feature sequences, which were then combined with the static features in a fully connected layer to predict CVD risk. We further proposed a phenotype-contrastive training strategy, i.e., the *Contrast_pheno_DL*, to enhance the model performance. To validate the effectiveness of the strategy, two other phenotype-specific DL models with different feature sets were implemented for comparison, i.e., *Pheno_spec_DL1* and *Pheno_spec_DL2*. The DL model architecture with different training strategies and feature sets are illustrated in (Fig. 3), and the details of the three DL models are described below:

- *Pheno_spec_DL1*: The feature set of this model includes all selected static features, the 29 PSG static features, and deep representations of the five sleep-event feature sequences.
- *Pheno_spec_DL2*: The feature set comprises the selected static features, the 29 PSG static features and sleep-event feature sequences that are specifically relevant to each phenotype. The aim is to focus on features that are directly related to each phenotype.
- *Contrast_pheno_DL*: This model utilizes the same features as the *Pheno_spec_DL1*, while its training approach is distinct in strategically merging different phenotypic populations that have distinct risk levels. The goal is to identify and learn discriminative features among various phenotypes.

#### 2.2.5 Performance Evaluation

In this study, subjects who experienced cardiovascular events were labeled as positive samples. The evaluation metrics include: Accuracy, Precision, Recall, F1-Score, Area under Curve of the Receiver Operating Characteristic Curve (AUC-ROC), Area under Curve of the Precision-Recall Curve (AUC-PRC).

#### 2.2.6 Feature Importance Analysis

The influence of all features on the model's predictive performance was evaluated by calculating the SHapley Additive exPlanation (SHAP) values derived from the field of cooperative game theory [30], to identify the key features for different phenotypes. The SHAP value of each feature was calculated according to the following equation:

(1) $$\emptyset_{i}=\sum_{S\subseteq F\backslash \left\{i\right\}}\frac{\left|S\right|!\left(\left|F\right|\;-\;\left|S\right|\;-\;1\right)!}{\left|F\right|!}\left[f_{S\cup \left\{i\right\}}\left(x_{S\cup \left\{i\right\}}\right)-f_{S}\left(x_{S}\right)\right]$$

in which, F represents the set of all features, S represents the subset of F that excludes feature i, $x_{S}$ represents the values of input features in the set S,and f represents the output value of the model.

## 3. RESULTS

### 3.1. Feature selection

51 features were selected by the lasso logistic regression with the alpha parameter set to 0.015. These features encompass not only conventional CVD risk factors like smoking, high-density lipoprotein cholesterol, and total cholesterol, but also extend to features from computed tomography (CT) and magnetic resonance imaging (MRI) ones. Additionally, they include a broad spectrum of other characteristics such as sleep patterns, anthropometry, dietary habits, cognitive status, and more. A detailed list of the selected features can be found in the Supplementary Table 1.

### 3.2 OSA phenotypes

3 and 6 OSA phenotypes were identified by the silhouette method and 4 by the elbow method on s all subjects, respectively. For 3 phenotypes, there was insufficient differentiation among the four distinct aspects of OSA pathophysiology, i.e., sleep architecture disturbances, autonomic dysregulation, respiratory disturbances, and hypoxemia [18–21]. In contrast, the division of 6 phenotypes was excessively granular, resulting in a lack of clear distinction between phenotypes. Categorizing these 29 features into 4 phenotypes effectively reflects various aspects of OSA pathophysiology, as shown in Supplementary Table 2. In each phenotype, the most prominent PSG features are highlighted in bold. Each phenotype was named according to its dominant pathophysiological domain: *Mild, Respiratory related, Sleep related, and Combined*.

#### Mild Phenotype:

Characterized by the lowest positive sample proportion of 6.7%, this phenotype exhibits the fewest respiratory events, highest sleep efficiency, near-normal sleep structure, and nighttime oxygen saturation levels (SpO_2_). It typically comprises healthy (Apnea Hypopnea Index, AHI < 5) to mild OSA (5 ≤AHI < 15) subjects.

#### Sleep Related Phenotype:

With the highest positive sample proportion of 15.1%, this phenotype presents significantly lower sleep efficiency, higher arousal and PLMS compared to the *Mild* type (*p*<0.001). It falls under moderate OSA (15 ≤ AHI < 30). The features of breathing disturbance and hypoxemia are less prominent compared to the *Respiratory* and *Combined* phenotypes.

#### Respiratory Related Phenotype:

This phenotype is characterized by significantly more frequent respiratory events, lower levels of nighttime SpO_2_ compared to the *Mild* type (*p*<0.001) and with moderate OSA. The features of sleep architecture disturbance and autonomic dysregulation are less prominent compared to the *Sleep related* and *Combined* phenotypes.

#### Combined Phenotype:

This type exhibits a mixture of the four pathophysiological domains—sleep architecture disturbance, autonomic dysregulation, breathing disturbance, and hypoxemia. It is classified as severe OSA (AHI ≥ 30).

As shown in (Table 1), the HRs for CVD events significantly differed among the four OSA phenotypes. Compared to the *Mild* phenotype, the *Respiratory*, *Sleep*, and *Combined* phenotypes show 2.708, 2.651, and 2.849 times higher risks to experience CVD events. Notably, the HRs, as determined by OSA phenotyping, does not align with the actual CVD event rates observed within each phenotype. This discrepancy suggests that additional factors are necessary to achieve more accurate predictions of CVD risk.

### 3.3 OSA Phenotyping based CVD Risk Prediction

**Figure 4:**
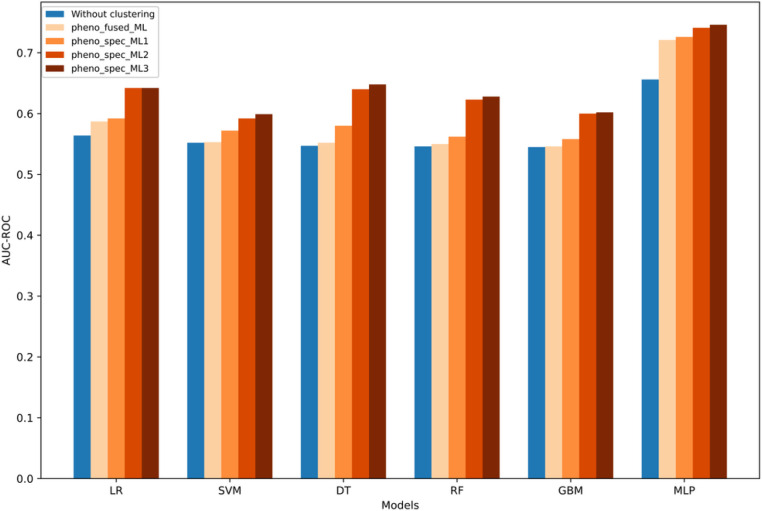
The AUC-ROC values of various ML models with and without OSA clustering.

As illustrated in (Fig. 4), the AUC-ROC values of various ML models were notably improved by incorporating OSA phenotyping for CVD risk prediction. Among all models, the MLP model achieved the best performance, with am AUC-ROC value of 0.656. Pheno_spec_ML3 presented the most significant improvement with the AUC-ROC value improved to 0.746. These findings underscore the importance of OSA phenotypic information in CVD risk prediction. A detailed comparison of model performance of various ML models with and without OSA clustering is shown in Table 3 in Supplementary material.

### 3.4 Integrating deep representation of feature sequences of sleep events

Table 2 shows the performance of three DL modeling approaches that incorporated OSA phenotypic information and deep representations of sleep-event feature sequences. There is a notable improvement for the two phenotype-specific models (*Pheno_spec_DL1* and *Pheno_spec_DL2*) when deep features were included as compared to *Pheno_spec_ML3*, with the AUC-ROC and AUC-PRC values exceeding 0.83 and 0.50, respectively. The *Contrast_pheno_DL* model, which involves contrastive training with a combination of the *Mild* phenotype and one of the other phenotypes (i.e., *Mild + Sleep, Mild + Respiratory, Mild + Combined*), achieved even higher AUC-ROC and AUC-PRC values of 0.877 and 0.689, respectively. These findings highlight the substantial additional value provided by overnight sleep-event feature sequences in predicting CVD risk across different OSA phenotypes.

Table 3 provides a detailed illustration of the performance of the DL models trained with specific (*Pheno_spec_DL2*) and contrastive phenotypes (*Contrast_pheno_DL*) across the four phenotypes. It reveals that combining the *Mild* phenotype with one of the other phenotypes generally improves model performance compared to the *Mild* phenotype alone across all phenotypes. Grouping the *Mild* phenotype with the *Combined* phenotype results in the most substantial improvement for the *Mild* Type.

### 3.5 Feature importance analysis

**Figure 5:**
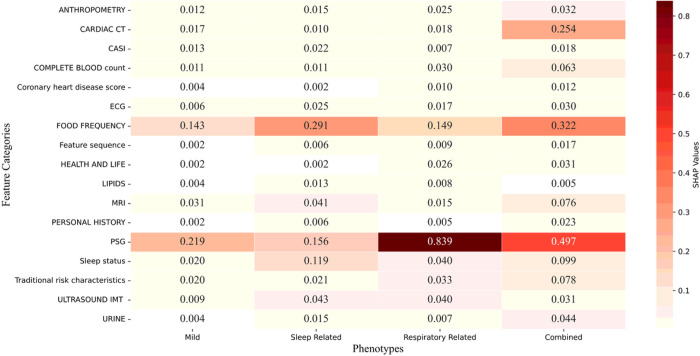
Heatmap of feature importance of the four phenotypes

As shown in Fig. 5, in addition to the traditional CVD risk features, PSG and FOOD FREQUENCY were recognized as very important features for all four phenotypes. Moreover, each phenotype placed emphasis on different additional features. Specifically, the *Sleep Related* phenotype particularly emphasized features reflecting features related to sleep status acqruied in the sleep questionnaire such as sleep duration, sleep efficiency standard deviation, etc. The *Combined* phenotype gave significant emphasis on CARDIAC CT features.

**Figure 6:**
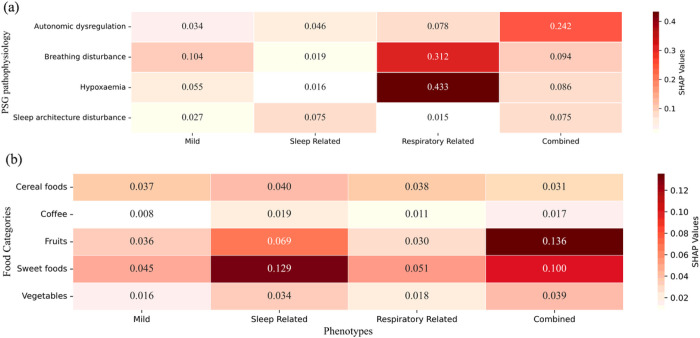
Distribution of importances to specific PSG and FOOD FREQUENCY features across the four phenotypes

Although all four phenotypes emphasized the PSG and FOOD FREQUENCY feature categories, they presented varying importance to specific PSG and FOOD FREQUENCY features. As shown in Fig. 6 (a), the *Mild* phenotype gave more emphasis on breathing disturbance features such as AHI. The *Sleep Related* phenotype focused on features related to sleep structure disorders, such as sleep efficiency and sleep duration. The *Respiratory* phenotype strongly emphasized blood oxygen saturation and AHI levels. The *Combined* phenotype specifically highlighted autonomic disorders features such as arousal and PLMS levels.

**Figure 7:**
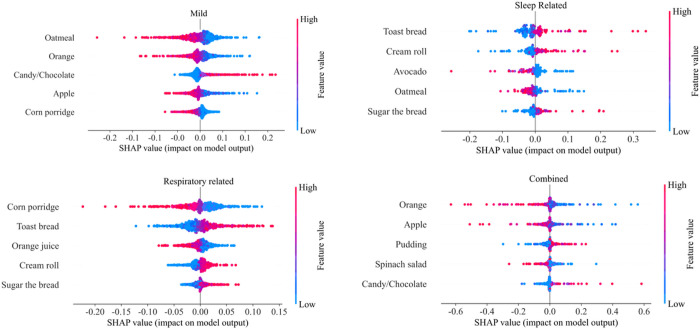
Top-five important foods of the four phenotypes

As shown in Fig. 6 (b), four phenotypes gave different emphasis to distinct FOOD FREQUENCY categories. Overall, the frequency of sweet food consumption is a relatively important risk factor for all phenotypes, especially for *Sleep* and *Combined* phenotypes. These two phenotypes all gave additional significant importance to fruits. *Sleep* and *Combined* phenotypic populations should pay special attention to sugar-controlled diets, and have more fruit intake. On the other hand, the importance distribution of foods in the *Mild* and *Respiratory Related* phenotypes is relatively similar, and the two groups should reduce sweets consumption and combine more grains and fruits intake. Fig. 7 further shows the top-five important foods on cardiovascular risk of the four phenotypes, which can serve as recommendations for daily dietary management.

## 4. DISCUSSION

Recent research has examined the association between OSA and CVDs, including phenotyping OSA using PSG static features and analyzing their differences in cardiovascular risks [13, 17, 34]. However, it remains to be explored on how to effectively using the OSA-related information for accurate CVD risk prediction modeling. Addressing this gap, this study built several ML and DL models under various OSA phenotyping integration strategies. Additionally, the study examined the value of integrating deep representations of sleep-event feature sequences on CVD risk prediction. The findings indicate that the approach based on OSA phenotyping and integrating deep representations of overnight sleep-event feature sequences, yield the most optimal performance in CVD risk prediction.

Regardless of the ML models employed, it is evident that performance improves significantly after incorporating phenotyping. Specifically, while the *Pheno_fused_ML* model, which integrates OSA phenotyping directly as a feature, shows some effectiveness. But, it is surpassed by the four phenotypes that were modeled separately (*Pheno_Spec_ML1*, *Pheno_Spec_ML2* and *Pheno_Spec_ML3*). This distinction could be attributed to the inability of *Pheno_fused_ML* model to adequately learn the weights of the phenotype feature amidst a multitude of other features. Furthermore, selecting phenotype-specific PSG static features to build the model further enhances the model performance (*Pheno_Spec_ML2* vs *Pheno_Spec_ML3*), a trend that was also observed in DL models (*Pheno_spec_DL2* vs *Pheno_spec_DL1*). The possible reason for this improved performance is that, features relevant to other phenotypes may introduce confounding factors in the risk prediction for a specific phenotype. By selecting PSG features tailored to specific phenotypes, models can more accurately learn and understand the relationship between these features and CVD risk within the context of that phenotype.

The risk prediction accuracy was further improved by adding the deep features from sleep-event sequences. In addition, we also found that the *Contrast_spec_DL* model achieved optimal performance by strategically combining one of the three OSA phenotypes (*Sleep-related, Breathing-related and Combined*) with the *Mild* phenotype, using all PSG static features and sleep event feature sequences as inputs, compared to a training strategy (i.e., *Pheno_spec_DL2*) that incorporated phenotype-specific sequence features. This approach led to a notable increase in the AUC-ROC value to 0.877, and improved predictive accuracy for each phenotype compared to their individual performances. This finding indicates that while modeling within a single phenotype generally yields better results than non-phenotyping approaches, strategic combination of samples from different phenotypes of different risk levels can enhance risk prediction for both groups. A possible explanation for this improvement is the relatively lower and less varied risk profiles within the *Mild* phenotype. When these were combined with a phenotype characterized by a broader variance in risk distribution, the model's capacity to discern relevant risk features was enhanced. Consequently, this study observed a significant improvement in the predictive performance for the *Mild* phenotype, especially when combined with the *Combined* phenotype, as indicated by a HR ranging from 1.566 to 6.424.

While previous studies have utilized ML or DL techniques to process short-term physiological sequences, focusing primarily on predicting specific CVD events [33, 38–41], this study first leveraged the temporal information from overnight sleep-event feature sequences through deep LSTM networks. It provides a more comprehensive set of features for CVD risk prediction than only using averaged static features. Given the objective to predict CVD risk over the next five years, the study recognized the highly complex temporal relationships exhibited by multiple physiological signals throughout the entire sleep period. Directly using these signals as model inputs might fail to capture essential information pertinent to long-term CVD risk. Consequently, the study focused on extracting temporal features from five key sleep-event feature sequences (arousal, hypopnea, apnea, hypoxemia and PLMS) over the full night.

Unlike previous studies that relied on manual selection of relevant risk factors for predictive modeling [35–37], this study employed a comprehensive feature selection process across all static features encompassing multi-dimensional aspects, including traditional risks factors, PSG-based multifaced factors, imaging markers and lifestyle factors, etc. This approach has the potential to lead to more integrated and precise predictions of CVD risk in clinical practice. Moreover, as far as we know, this is the first study to rank the importance of features among a comprehensive categories of cardiovascular risk features. One important finding is that, the four OSA phenotypes all gave particular emphasis on PSG and FOOD FREQUENCY features. This is consistent with existing understanding on the importance of PSG features for CVD risk prediction in the literature [42–44]. Earlier studies also highlighted the close association between dietary habits and CVD risk [45–48]. Through the comprehensive analysis of feature importance in this study, the importance of dietary habits in predicting CVD risk should be further emphasized in the general population. Additionally, each phenotype emphasized distinct PSG and FOOD FREQUENCY features. These findings would enable more precisive CVD risk evaluation and management for different OSA phenotypes.

Despite these promising results, the study has some limitations. It utilized data from only 1,874 participants from the MESA dataset, and only 187 subjects were used in the testing set. The generalization capability of the model needs further validation on larger datasets. Additionally, the model depended on sleep-event annotation based on the PSG data, which was manually labeled, a resource-intensive and time-consuming process. However, considering ongoing research efforts in developing automated sleep staging and event detection algorithms [49, 50], future work could leverage fully automated algorithms to provide the input features required for this model. Future studies may also investigate the minimally effective set of features that maintains the performance of CVD risk prediction with the strategies proposed in this study.

## 5. CONCLUSIONS

This study introduces a new approach for CVD risk prediction, which integrates deep features learned from overnight sleep-event feature sequences. Additionally, the study validated the superior performance of OSA phenotype-specific models over phenotype-agnostic ones, and also introduces a training method that strategically combines the *Mild* phenotype with another OSA phenotypic population (*Respiratory, Sleep* or *Combined*) for contrastive training. The contrastive phenotype-specific model with deep features achieved an accuracy of 96.6% and an AUC-ROC value of 87.7% in predicting CVD risk over five years in general population without historical CVDs. Moreover, the development of phenotype-aware predictive models provided valuable insights into key risk features. The model placed a significant emphasis on lifestyle-related features such as the sleep factors indicated by PSG and food habits, over traditional and other risk factors for predicting long-term CVD outcomes in the general population. Furthermore, each of the four phenotypes emphasized distinct features, which may pave the way for precise risk management strategies tailored for different OSA phenotypic populations. Future research should validate these findings on additional datasets, and explore the utility of mobile and wearable devices for regularly collecting physiological and lifestyle data over extended periods, which could offer a more accurate representation of lifestyle information, potentially providing early warning of CV risks.

## Figures and Tables

**Table 1:** Cox hazard ratios for CVD events of four OSA phenotypes

Parameters	HR	95% CI	*P*-value
OSA phenotypes			

Mild	Reference		

Respiratory related	2.708	1.913–3.888	0.003

Combined	2.849	1.566–6.424	0.008

Sleep related	2.651	1.901–4.714	0.001

**Table 2:** Results of different CVD risk prediction models based on the MESA dataset

Related studies						
Model	Number of subjects (Proportion of positive subjects, %)		Research Objectives			AUC-ROC
MES-C risk score [31]	632 8.2%		Predictive value of atherogenic calcium score for 10-year risk of CVD			0.77
PCP-HFCKD risk equation [32]	2328 14.6%		Patients with chronic kidney disease_10-year Heart Failure prediction			0.79
DeepSurv [33]	6814 -		One-year Risk Prediction for Atherosclerotic Cardiovascular Disease			0.82
Our study						
Model	Accuracy	Precision	Recall	F1-score	AUC-PRC	AUC-ROC
*Pheno_spec_ML3*	0.933	0.736	0.446	0.555	0.496	0.746
*Pheno_spec_DL1*	0.920	0.579	0.611	0.595	0.507	0.832
*Pheno_spec_DL2*	0.943	0.686	0.608	0.645	0.573	0.839
***Contrast_pheno_DL***	**0.966**	**0.851**	**0.750**	**0.797**	**0.689**	**0.877**

**Table 3:** The performance of the DL models across the four phenotypes trained with specific (*pheno_spec_DL2*) and contrastive phenotypes (*contrast_pheno_DL*)

AUC-ROC /AUC-PRC	*Pheno_spec_DL2*	*Contrast_pheno_DL*		
		Mild + Sleep	Mild + Respiratory	Mild + Combined
Mild	0.750/0.534	0.785/0.590	0.833/0.619	**0.833/0.689**
Sleep related	0.915/0.808	**0.915/0.810**	-	-
Respiratory related	0.732/0.350	-	**0.854/0.567**	-
Combined	0.958/0.600	-	-	**0.979/0.750**
